# Breastfeeding and the Developmental Origins of Asthma: Current Evidence, Possible Mechanisms, and Future Research Priorities

**DOI:** 10.3390/nu10080995

**Published:** 2018-07-30

**Authors:** Kozeta Miliku, Meghan B. Azad

**Affiliations:** 1 Manitoba Developmental Origins of Chronic Diseases in Children Network (DEVOTION), Children’s Hospital Research Institute of Manitoba, University of Manitoba, Winnipeg, MB R3E 3P4, Canada; Kozeta.Miliku@umanitoba.ca; 2 Department of Pediatrics and Child Health, University of Manitoba, Winnipeg, MB R3E 3P4, Canada

**Keywords:** asthma, wheezing, breastfeeding, human milk, breast milk, infant nutrition, developmental origins of health and disease, developmental programming

## Abstract

Breastfeeding has many established health benefits, but its impact on asthma development is uncertain. Breastfeeding appears to have a positive and dose-dependent impact on respiratory health, particularly during early childhood and in high-risk populations; however, the strength and causality of these associations are unclear. It is challenging to compare results across studies due to methodological differences and biological variation. Resolving these inconsistencies will require well-designed, prospective studies that accurately capture asthma diagnoses and infant feeding exposures (including breastfeeding duration, exclusivity, and method of feeding), account for key confounders, evaluate dose effects, and consider effect modification and reverse causality. Mechanistic studies examining human milk bioactives and their impact on lung health and asthma development are beginning to emerge, and these will be important in establishing the causality and mechanistic basis of the observed associations between breastfeeding and asthma. In this review, we summarize current evidence on this topic, identify possible reasons for disagreement across studies, discuss potential mechanisms for a causal association, and provide recommendations for future research.

## 1. Introduction

Asthma is a lifelong disease with origins in early life. It is the most common chronic health problem in childhood, affecting 14% of school-aged children [1]. Asthma is a major cause of school absenteeism and pediatric hospitalization [2], placing a considerable burden on children, families, and society [3]. Multiple lines of evidence suggest a critical role for environmental exposures during early life, since asthma is frequently established in early childhood [4]. The developmental origins of health and disease (DOHaD) hypothesis proposes that nutritional and environmental exposures can “program” metabolic and immune development during critical periods of early life, inducing permanent long-term changes in physiology and affecting susceptibility to chronic diseases [5,6,7] including asthma [4,8]. For example, prenatal and early postnatal exposure to antibiotics, mold, tobacco smoke, and air pollution have all been associated with asthma development [4]. In addition, frequent lower respiratory infections are associated with wheezing during the first year of life, and asthma later in childhood [9].

**Figure 1:**
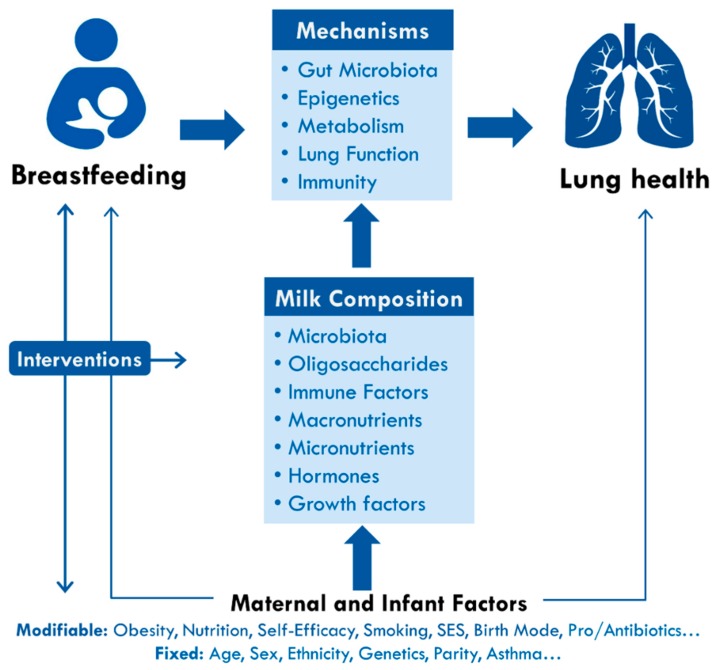
Breastfeeding and the developmental origins of lung health and childhood asthma. Breastfeeding may be associated with lung heath through several potential mechanisms, including modulation of gut microbiota, epigenetics, immunity, and lung development. These processes are driven by multiple bioactive components of human milk, which are influenced by various fixed and modifiable maternal and infant factors. Understanding these mechanisms and associations will help to inform interventions to improve lung health and reduce asthma risk by supporting breastfeeding and optimizing milk composition and infant nutrition. SES, socioeconomic status.

Infant feeding is another important early-life exposure that may influence respiratory infections and the developmental programming of asthma (Figure 1). The World Health Organization (WHO) recommends early initiation of breastfeeding within 1 h of birth, exclusive breastfeeding for the first six months of life, and continued breastfeeding up to two years of age or beyond [10,11]. In addition to providing optimal nutrition to support infant growth, human milk is an immunologically complex fluid containing multiple components that promote the development of innate and adaptive immunity [12,13]. In the Canadian Healthy Infant Longitudinal Development (CHILD) Study, we have observed that breastfeeding is associated with lower rates of wheezing in the first year of life [14] and lower odds of possible or probable asthma by three years of age [15], consistent with other cohorts in Canada, Sweden, United States and Australia [16,17,18]. However, not all studies have confirmed this association [19,20,21].

The controversy surrounding breastfeeding and atopic conditions, including asthma, dates back to 1936 when Grulee and Sanford reported a protective association between breastfeeding and eczema [22]. In a recent meta-analysis, Dogaru et al. reported a protective association in early childhood, with effects diminishing over time; however, pooled assessments were limited by individual study quality and methodology issues, resulting in considerable heterogeneity [23]. In the current review, our objectives are (1) to identify possible reasons for the conflicting evidence regarding breastfeeding and asthma development; (2) to discuss potential mechanisms for a causal association; and (3) to provide recommendations for future research.

## 2. Breastfeeding and Asthma: What is the Evidence, and Why Is It Inconsistent?

The impact of breastfeeding on lung function and asthma development is controversial [23,24,25,26]. Some studies report evidence or tendencies for beneficial effects from breastfeeding [16,17,18,27], but others have found no association or even an increased risk of asthma in breastfed children [19,20,21]. Several systematic reviews have been conducted to synthesize the existing evidence on this topic [23,24,25]. Dogaru et al. [23] reviewed 117 studies and found that “more versus less breastfeeding” was associated with a 22% reduced risk of asthma (pooled OR 0.78, 95% CI 0.74, 0.84), with the strongest effects being observed before two years of age, when asthma diagnosis is challenging to confirm [28], as discussed below. In another meta-analysis of 29 studies reporting childhood asthma from five to 18 years, Lodge et al. [24] found a 10% reduced risk (pooled OR 0.90, 95% CI 0.84, 0.97), with stronger associations in low- and middle-income countries. In both reviews, significant heterogeneity was observed (*I*^2^ = 71% and 63%, respectively), and the overall quality of evidence was considered to be low, with serious limitations. Below, we describe the major issues and possible explanations for the apparently inconsistent evidence regarding breastfeeding and asthma development across different studies.

### 2.1. Breastfeeding Definitions

Infant feeding can be measured and described in many ways, making it difficult to compare results across studies [29]. Various terminologies and criteria are inconsistently applied to describe exclusive, full, predominant, or partial breastfeeding. The WHO [11] defines exclusive breastfeeding as feeding human milk only (including donor human milk), without any food, water, or other fluids, although vitamin and mineral supplements or medicine syrups are allowed. Most studies do not capture sufficient information to apply this definition, and many do not document the total duration of breastfeeding (infant age at weaning); hence, systematic reviews have been limited to comparing “ever versus never” breastfeeding or “more versus less” breastfeeding [23,24]. Without information on the exclusivity and duration of breastfeeding, it is not possible to examine “dose effects”, which are helpful in assessing causality. In the CHILD Study, we have observed a dose-dependent protective association between breastfeeding and infant wheezing in the first year of life [14], as well as possible or probable asthma by age 3 years [15], with stronger associations for exclusive and sustained breastfeeding.

Aside from breastfeeding exclusivity and duration, other potentially important details of infant feeding exposures are often ignored in epidemiologic studies. Few consider the method of breast milk feeding (directly at the breast versus pumped and bottled), the type of complementary feeding in partially breastfed infants (formula versus solid or semisolid foods), the relative proportion of breast milk compared to other sources of nutrition, or the use of donor milk. These details may be relevant to asthma research. For example, we recently found that supplementing breast milk with infant formula appears to diminish its protective effect against wheezing during infancy, whereas supplementing with solid foods did not [14]. Extended analyses later in childhood and replication in other cohorts will be required before the relevance to asthma can be determined.

In terms of feeding mode, we and others have reported that feeding at the breast appears to be more protective than breast milk fed from a bottle [15,30]. The reason for this difference remains to be determined, but it is possible that bioactive components of milk may be altered during the expression and storage of breast milk [31,32,33] or that expressed breast milk contains asthmogenic chemicals or contaminants from breast pumps or storage containers [34,35]. Suckling at the breast may also function as “physical exercise” to stimulate lung growth [36] and provide exposure to protective maternal skin microbes. Regardless of the mechanism, these findings indicate that feeding mode is important to consider in breastfeeding research, yet it is rarely reported. Similarly, the use of pasteurized human donor milk is increasing [37], but it is rarely reported or studied in relation to respiratory health. This is relevant because many bioactive components of human milk are compromised or destroyed during pasteurization. As the use of pumped milk and donor milk increases, it will be important to document these exposures and study their impact on immunological outcomes in the infant.

### 2.2. Asthma Definitions

Asthma is a highly variable phenotype that is inconsistently defined across studies. In their meta-analysis, Dogaru et al. evaluated three definitions, including “asthma ever” (from a variety of sources including medical records, parent reports of doctor diagnosis, use of asthma medication, or wheeze accompanied by bronchial hyperreactivity); “recent asthma” (“asthma ever” criteria within the last 12 months); and “recent wheezing illness” (“recent asthma” or any episodes of wheezing in the last 12 months). Clinically, asthma is defined as a chronic inflammatory disorder of the lungs that is characterized by recurrent episodes of wheezing, shortness of breath, chest tightness, and coughing that vary over time and in intensity, together with a limitation of expiratory airflow [38]. Lung function testing is challenging to perform before the age of five years [39], making it difficult to diagnose asthma in young children [28], yet many studies report this outcome. Indeed, Dogaru et al. found the strongest protective association of breastfeeding and “asthma” among studies evaluating children under two years of age, with effects apparently diminishing over time [23]. Consistent with this finding, the prospective population-based Generation R Study reported a protective association of breastfeeding and asthma at six years of age [27], but this association diminished by the age of 10 years [40].

Many studies do not measure lung function and must rely on parent-reported or self-reported asthma symptoms or medication use. This is problematic because wheezing is often used as the diagnostic marker of asthma, yet wheezing can be caused by infections that are unrelated to asthma, and not all infants or children who wheeze proceed to develop asthma [41]. In addition, different asthma phenotypes exist, such as atopic or non-atopic asthma, and early- or late-onset asthma [42,43]. Recent data from the large population-based UK Biobank study suggests that breastfeeding may have a different direction of effect on non-atopic asthma compared with atopic asthma [44], being protective only for the former. Thus, a precise and accurate definition of asthma is important because different asthma phenotypes may be differentially associated with breastfeeding and other early-life exposures [15].

### 2.3. Study Design Issues and Limitations

Randomized controlled trials provide the best evidence of causal effects, but this approach cannot be directly applied to breastfeeding research because it is unethical to randomize breastfeeding; hence clinical trials are lacking in this field. Randomization has been indirectly applied to breastfeeding in the Promotion of Breastfeeding Intervention Trial (PROBIT) [45], where maternity hospitals in Belarus were randomly assigned to receive a breastfeeding promotion intervention. Women delivering at intervention versus control hospitals had substantially higher rates of exclusive breastfeeding at three months (43% versus 6%) and slightly longer durations of breastfeeding (20% versus 11% still breastfeeding at 12 months) [45]. Among many health outcomes, asthma was evaluated in secondary analyses at the ages of 6.5 (parent-reported) and 16 years (self-reported) [46,47], with no significant differences observed at either time point (OR 1.2; 95% CI 0.7, 1.9 at 6.5 years; OR 0.8; 95% CI 0.5, 1.2 at 16 years) [46,47]. These null findings suggest that breastfeeding does not affect asthma development; however, the analyses were limited by the subjective questionnaire-derived measure of asthma, and they lacked power due to the relatively low prevalence of asthma in Belarus (just 1.6% compared to the worldwide average of 14%) [47,48]. It is also possible that a protective effect of breastfeeding against asthma was not detectable in this trial because of the relatively minor impact of the PROBIT intervention on sustained breastfeeding.

Because it is generally not feasible to randomize breastfeeding, the majority of evidence on this topic comes from observational studies, where it can be challenging to interpret results related to infant feeding [49]. Observational studies vary greatly in their methodology for capturing and defining feeding exposures and asthma-related outcomes (discussed above), and they are subject to confounding and recall bias [50] (discussed below); all of these factors have likely contributed to the high heterogeneity reported in meta-analyses of observational studies on breastfeeding and asthma [10,23,24].

### 2.4. Confounding

Observational studies vary in their ability and approaches to address confounding bias. Confounding occurs when the exposure and outcome in question are both associated with a third a factor (confounder), causing a spurious association. Socioeconomic status and lifestyle are important confounders in breastfeeding research because breastfeeding is often (though not always [51,52]) socially patterned, and socioeconomic factors can influence asthma development through multiple mechanisms other than breastfeeding. In the CHILD Study [14,15,53] and other contemporary Western populations [54,55,56], breastfeeding rates are lower among mothers with lower incomes and education, mothers who smoke, and mothers who are obese. These maternal characteristics are also independent risk factors for childhood asthma, so it is necessary to document and control for them before estimating the independent contribution of breastfeeding. Even when multiple confounders are accounted for, it is possible that residual (unmeasured) confounding exists. This can be addressed using alternative approaches to minimize residual confounding, such as sibling controls [57], cross-cohort comparisons, or Mendelian randomization [58].

### 2.5. Reverse Causality

Reverse causality is another potential source of bias in observational research. This occurs when the outcome precedes and causes a change in the exposure (i.e., causality occurs in the reverse direction). In the case of asthma and breastfeeding, it is possible that a mother noticing early signs of asthma in her infant (e.g., wheezing) might be motivated to extend breastfeeding, which could mask a protective effect, or even result in a false negative association [59]. Longitudinal observational studies with repeated prospective measures of infant feeding and respiratory health are required in order to determine the temporality of feeding exposures relative to asthma development. This design facilitates analyses to address confounding by reverse causation—for example, in sensitivity analyses excluding infants who developed asthma symptoms prior to breastfeeding cessation.

### 2.6. Effect Modification

Effect modifiers are factors that alter the association between the exposure and outcome under investigation. Failing to account for this interaction may obscure or exaggerate a true association. In the CHILD study we found that maternal asthma and infant sex modify the association of breastfeeding and wheezing. We observed a dose-dependent protective association among infants born to mothers with asthma [14], especially for boys, while there was no association in the absence of maternal asthma, where wheezing rates were much lower overall [14]. Similar findings were reported from the Leicestershire Cohort Studies from Dogaru et al. where breastfeeding for more than four months was associated with superior lung function (increased forced midexpiratory flow) in children of mothers with asthma [60]. Other established asthma risk factors that could be mitigated by breastfeeding include exposures that alter the gut microbiota (which is strongly influenced by human milk—see Section 3.2), including cesarean delivery, antibiotics, and day care attendance [4]. It has also been reported that associations of air pollution and respiratory conditions were minimized among children who were breastfed, suggesting that breastfeeding reduces susceptibility to the respiratory effects of pollutants [61]. Breastfeeding may also minimize risks associated with passive smoking [62] and psychosocial stress [63]. Together, this evidence suggests that breastfeeding might be particularly beneficial among high-risk infants with a genetic predisposition to asthma and/or an “asthmogenic” environment in early life. This could explain why studies in low-risk populations do not consistently observe an association between breastfeeding and asthma. Since many of the above factors could also be confounders or mediators of the association between breastfeeding and asthma, it is important to use appropriate statistical methods to evaluate confounding, mediation and effect modification.

### 2.7. Study Settings and Breastfeeding Culture

Variations in study populations and settings also contribute to heterogeneity across studies. Asthma prevalence and etiology differs between countries [1,64,65], and breastfeeding may differentially affect different asthma phenotypes [44]. Indeed, a recent meta-analysis found that the protective effects of breastfeeding are more noticeable in low to middle-income countries where children are at greater risk of severe respiratory infections [24]. Breastfeeding practices also vary substantially across settings due to differences in breastfeeding “culture” and policies [66,67,68], potentially leading to a different impact on respiratory health. In some populations, there may be a “social prestige” associated with formula feeding [69], despite the recognized risks and potential for contamination in settings without access to clean water [70]. Countries also vary in their implementation of maternity leave policies and the WHO Code of Marketing of Breastmilk Substitutes, both of which can significantly impact the initiation, duration, and method of breastfeeding [71,72]. In settings without paid maternity leave, such as the US, the majority of infants are fed pumped milk [73], thus “breastfeeding” is likely to be “bottled breast milk” which may have different effects on infant health and asthma risk [15]. Finally, the prevalence of key confounders and effect modifiers, (e.g., maternal asthma, smoking, antibiotic use, cesarean sections) can differ substantially between countries. All of these setting-specific factors may differentially influence mothers’ desire and ability to breastfeed, as well as the “effectiveness” of breastfeeding and the confounding structures that must be considered in different settings.

### 2.8. Human Milk Composition

In addition to variations in study settings and methodology (discussed above), variations in human milk composition may also contribute to the conflicting results obtained from breastfeeding studies in different settings and populations. Human milk is complex and personalized, containing micro- and macro-nutrients, oligosaccharides, cytokines, enzymes, growth factors, immune cells, and microbes. These bioactive components influence the maturation of the infant gut microbiota and immune system [13,74], which can subsequently influence asthma development [75]. Systematic reviews have found emerging, though still inconclusive evidence that human milk fatty acids [76], oligosaccharides [77], and TGF-beta [78] may influence immunological outcomes in breastfed infants. The concentration of these and other bioactive factors in human milk is highly variable, and can be affected by multiple genetic and environmental factors that differ at the individual and population levels [79,80,81] (Figure 1), including ethnicity, diet, body composition, smoking, immunization history, health status (e.g., asthma, allergies), geographic location, and method of delivery [82]. Differences in these milk-modifying factors may explain why breastfeeding appears to have different effects in different populations. For example, we [83] and others [84,85,86] have observed that milk fatty acid composition varies widely (up to 20-fold) [87] by geographic location and ethnicity, and intervention studies show that fish consumption can increase levels of human milk docosahexaenoic acid (DHA) [88], which may influence asthma development [89]. Thus, breastfeeding effects may be stronger in coastal populations with high fish consumption, where breastfed infants receive more DHA in their mothers’ milk. Similarly, probiotic use differs widely between countries and may influence the cytokine profile of human milk [90], which could modify the effect of breastfeeding on asthma development.

## 3. Mechanisms: How Could Breastfeeding Protect Against Asthma?

There are several plausible explanations for a causal association between breastfeeding and asthma, involving epigenetic effects, modulation of gut microbiota, and stimulation of lung growth and immune development (Figure 1).

### 3.1. Epigenetics

Epigenetic alterations in gene expression are a commonly suggested mechanism for developmental programming, where early-life exposures have a lasting impact on later health outcomes [91]. Epigenetics has also been linked specifically to allergies and asthma [92]. Emerging evidence suggests that breastfeeding may affect epigenetic programming in breastfed infants by influencing DNA methylation [93], although relatively few studies have explored this hypothesis. Breastfeeding has been inversely associated with promoter methylation of the appetite-regulating *LEP* gene [64] and the tumor suppressor *CDKN2A* gene [94]. In addition, breastfeeding has been associated with overall patterns of DNA methylation [95]. Moreover, breastfeeding may have epigenetic effects beyond DNA methylation, since human milk contains non-coding RNAs that regulate gene expression [96,97]. Together, these studies suggest that breastfeeding may influence epigenetics, but further research is needed to confirm these effects and determine their relevance to the association of breastfeeding and asthma.

### 3.2. Microbiota

Multiple studies [98,99] have shown that breastfeeding profoundly influences the development of the infant oral and gut microbiota [100], which have been independently linked with asthma development [75]. Gut microbiota, in particular, play an important role in training the naïve infant immune system [101]. Human milk contains live microbes that help seed the infant gut, as well as human milk oligosaccharides (HMOs) that provide a selective substrate for gut microbiota [102]. The direct skin-to-skin contact during breastfeeding may provide an additional source of protective maternal microbes to the nursing infant [103]. Commercial infant formulas do not contain the diverse and personalized prebiotic and probiotic components found in human milk, and thus cannot optimally support the natural assembly and development of the human gut microbiota, which may lead to altered immune development and increased susceptibility to asthma in later life among formula-fed infants [104]. While several studies have observed associations between gut microbiota during infancy and asthma later in childhood [75,104], few have addressed the contribution of human milk components.

### 3.3. Immunity and Inflammation

Breastfeeding modulates the development of the infant mucosal and systemic immune systems. Human milk contains numerous immunomodulators (a-tocopherol, b-casomorphins, prolactin, soluble toll-like receptor 4) and anti-inflammatory agents (lactoferrin, lysozyme, antioxidants, cytokines, secretory IgA) [13]. Breastfed infants appear to have superior immune function compared to formula-fed infants, with an enhanced capacity to mount a targeted response to potential pathogens [105]. Consistent with this evidence, breastfed children have fewer respiratory tract infections in early life [9]; this may contribute to the protective effect of breastfeeding against asthma development since lower respiratory tract infections are an established risk factor for asthma [27]. In addition, breastfeeding promotes maternal-infant attachment, which positively impacts cortisol regulation in breastfed infants [106], and may subsequently prevent chronic inflammation and asthma risk [107].

### 3.4. Lung Growth and Pulmonary Function

Breastfeeding has been shown to support lung growth and enhance lung function [60], suggesting a protective effect on asthma risk. Ogbuanu et al. showed that breastfed children have increased lung volumes by the age of 10 years, and attributed this advantage to the mechanical stimulus associated with suckling at the breast in early life [36]. In their systematic review of 10 studies, Waidyatillake et al. provide further evidence that breastfeeding is beneficial for lung function, and propose that effects may be mediated by reduced infections and greater height in breastfed children [108]. The authors also note that only a few studies have explored whether maternal asthma might modify the effect of breastfeeding on lung function, with inconsistent results.

## 4. Conclusions and Recommendations

Breastfeeding has many established short- and long-term benefits for maternal and child health, including protection from infections and enhanced neurodevelopment during infancy, reduced risk of obesity later in childhood, and reduced risk of type 2 diabetes and breast cancer among mothers who breastfeed [10]. However, the impact of breastfeeding on asthma remains unclear. This uncertainty is reflected in the inconsistent results from many previous studies with significant methodological limitations and is likely also related to the tremendous biological variability in human milk. Further epidemiologic and biomedical research is needed to clearly define the association of breastfeeding and asthma development, to establish causality, and to characterize the underlying biological mechanisms.

Recommendations for future research in new studies, or using existing datasets, are summarized in Table 1. Because randomized control trials are not feasible for studying breastfeeding, longitudinal prospective birth cohorts with repeated exposure and outcome assessments are the preferred study design. Standardized and consistent definitions for both breastfeeding and asthma should be applied. It is important to evaluate “dose effects” according to breastfeeding exclusivity and duration, and if possible, to document the method of feeding and type of complementary nutrition. Key confounders must be taken into account (e.g., socioeconomic status, maternal smoking) and potential effect modifiers should be examined (e.g., maternal asthma, infant sex, microbiota-modifying exposures). Alternative approaches to address confounding factors can also be employed (e.g., sibling controls, cross-cohort comparisons, Mendelian randomization), and Hill’s criteria on causation should be considered [109]. These criteria include the strength, consistency, temporality, and biological plausibility of observed associations, as well as supporting experimental evidence from mechanistic studies. Finally, external validity (generalizability) should be carefully considered and communicated.

In order to establish causality and uncover biological mechanisms, new research approaches are needed. For example, it is unclear whether maternal or infant genetics can modify the health effects of breastfeeding, and the potential epigenetic effects of breastfeeding are poorly understood. In addition, relatively little is known about the individual and collective impact of specific human milk components on lung development and asthma risk. Multi-omic and machine learning approaches may be required to account for the complex nature of human milk. Ultimately, research in these domains has the potential to inform new evidence-based strategies for asthma prevention, including initiatives to support breastfeeding, interventions to “optimize” human milk composition, and alternative nutrition-based strategies for infants who cannot be breastfed.

## Figures and Tables

**Table 1:** Recommendations for future research on breastfeeding and asthma in new studies or using existing datasets.

Domain	Recommendations
Study design	Ideally, recruit prenatally and follow prospectively
Breastfeeding exposures	Essential: capture exclusivity and duration, and evaluate dose effects: breastfeeding duration as a continuous measurement duration of exclusive breastfeeding duration of any breastfeeding Ideally, define using international criteria [29] and distinguish between: feeding at the breast versus bottled breast milk donor human milk versus mother’s own milk mixed feeding with formula versus solid foods relative proportion of breast milk versus other nutrition
Asthma outcomes	Define using international criteria [38,110]. Ideally, use objective physician diagnosis and capture pulmonary function. Also consider: asthma phenotypes (e.g., atopic versus non-atopic) age of onset (diagnosis is uncertain before age 5)
Confounding	Adjust for essential confounders socioeconomic status maternal smoking gestational age Consider additional approaches to address confounding sibling controls Mendelian randomization cross-cohort comparisons
Effect Modifiers	Test for interactions or conduct stratified analyses by: maternal asthma maternal and infant genetics infant sex microbiota-disrupting factors (c-section, antibiotics) preterm birth exposure to smoke/pollution
Reverse causality	Conduct sensitivity analyses excluding infants with asthma symptoms prior to weaning.
Mechanistic studies	Use biological specimens or pre-clinical models to study causal pathways identified in clinical studies epigenetics bioactive components of human milk (integrated multi-omic approaches) immunity microbiota lung growth
Generalizability	Consider and comment on generalizability of results based on study population and setting (e.g., breastfeeding rates, policies and culture; asthma prevalence)

## References

[1] Pearce N., Ait-Khaled N., Beasley R., Mallol J., Keil U., Mitchell E., Robertson C. (2007). Worldwide trends in the prevalence of asthma symptoms: Phase III of the international study of asthma and allergies in childhood (ISAAC). Thorax.

[2] Harrison B.D., Pearson M.G. (1993). Audit in acute severe asthma––Who benefits?. J. R. Coll. Physicians Lond..

[3] Barnett S.B., Nurmagambetov T.A. (2011). Costs of asthma in the United States: 2002–2007. J. Allergy Clin. Immunol..

[4] Kozyrskyj A.L., Bahreinian S., Azad M.B. (2011). Early life exposures: Impact on asthma and allergic disease. Curr. Opin. Allergy Clin. Immunol..

[5] Barker D.J. (1990). The fetal and infant origins of adult disease. BMJ.

[6] Wadhwa P.D., Buss C., Entringer S., Swanson J.M. (2009). Developmental origins of health and disease: Brief history of the approach and current focus on epigenetic mechanisms. Semin. Reprod. Med..

[7] Gluckman P.D., Hanson M.A., Beedle A.S. (2007). Early life events and their consequences for later disease: A life history and evolutionary perspective. Am. J. Hum. Biol..

[8] Duijts L., Reiss I.K., Brusselle G., de Jongste J.C. (2014). Early origins of chronic obstructive lung diseases across the life course. Eur. J. Epidemiol..

[9] Oddy W.H. (2004). A review of the effects of breastfeeding on respiratory infections, atopy, and childhood asthma. J. Asthma.

[10] Victora C.G., Bahl R., Barros A.J.D., Franca G.V.A., Horton S., Krasevec J., Murch S., Sankar M.J., Walker N., Rollins N.C. (2016). Breastfeeding in the 21st century: Epidemiology, mechanisms, and lifelong effect. Lancet.

[11] World Health Organization Indicators for Assessing Infant and Young Child Feeding Practices: Part 1 Definitions: Conclusions of a Consensus Meeting. http://apps.who.int/iris/bitstream/handle/10665/43895/?sequence=1.

[12] Greer F.R., Sicherer S.H., Burks A.W. (2008). Effects of early nutritional interventions on the development of atopic disease in infants and children: The role of maternal dietary restriction, breastfeeding, timing of introduction of complementary foods, and hydrolyzed formulas. Pediatrics.

[13] Munblit D., Peroni D.G., Boix-Amoros A., Hsu P.S., Land B.V., Gay M.C.L., Kolotilina A., Skevaki C., Boyle R.J., Collado M.C. (2017). Human milk and allergic diseases: An unsolved puzzle. Nutrients.

[14] Azad M.B., Vehling L., Lu Z., Dai D., Subbarao P., Becker A.B., Mandhane P.J., Turvey S.E., Lefebvre D.L., Sears M.R. (2017). Breastfeeding, maternal asthma and wheezing in the first year of life: A longitudinal birth cohort study. Eur. Respir. J..

[15] Klopp A., Vehling L., Becker A.B., Subbarao P., Mandhane P.J., Turvey S.E., Lefebvre D.L., Sears M.R., Azad M.B. (2017). Modes of infant feeding and the risk of childhood asthma: A prospective birth cohort study. J. Pediatr..

[16] Dell S., To T. (2001). Breastfeeding and asthma in young children: Findings from a population-based study. Arch. Pediatr. Adolesc. Med..

[17] Kull I., Almqvist C., Lilja G., Pershagen G., Wickman M. (2004). Breast-feeding reduces the risk of asthma during the first 4 years of life. J. Allergy Clin. Immunol..

[18] Oddy W.H., Holt P.G., Sly P.D., Read A.W., Landau L.I., Stanley F.J., Kendall G.E., Burton P.R. (1999). Association between breast feeding and asthma in 6 year old children: Findings of a prospective birth cohort study. BMJ.

[19] Burgess S.W., Dakin C.J., O’Callaghan M.J. (2006). Breastfeeding does not increase the risk of asthma at 14 years. Pediatrics.

[20] Wright A.L., Holberg C.J., Taussig L.M., Martinez F.D. (2001). Factors influencing the relation of infant feeding to asthma and recurrent wheeze in childhood. Thorax.

[21] Sears M.R., Greene J.M., Willan A.R., Taylor D.R., Flannery E.M., Cowan J.O., Herbison G.P., Poulton R. (2002). Long-term relation between breastfeeding and development of atopy and asthma in children and young adults: A longitudinal study. Lancet.

[22] Grulee C.G., Sanford H.N. (1936). The influence of breast and artificial feeding on infantile eczema. J. Pediatr..

[23] Dogaru C.M., Nyffenegger D., Pescatore A.M., Spycher B.D., Kuehni C.E. (2014). Breastfeeding and childhood asthma: Systematic review and meta-analysis. Am. J. Epidemiol..

[24] Lodge C.J., Tan D.J., Lau M.X., Dai X., Tham R., Lowe A.J., Bowatte G., Allen K.J., Dharmage S.C. (2015). Breastfeeding and asthma and allergies: A systematic review and meta-analysis. Acta Paediatr..

[25] Brew B.K., Allen C.W., Toelle B.G., Marks G.B. (2011). Systematic review and meta-analysis investigating breast feeding and childhood wheezing illness. Paediatr. Perinat. Epidemiol..

[26] Gdalevich M., Mimouni D., Mimouni M. (2001). Breast-feeding and the risk of bronchial asthma in childhood: A systematic review with meta-analysis of prospective studies. J. Pediatr..

[27] Den Dekker H.T., Sonnenschein-van der Voort A.M., Jaddoe V.W., Reiss I.K., de Jongste J.C., Duijts L. (2016). Breastfeeding and asthma outcomes at the age of 6 years: The generation R study. Pediatr. Allergy Immunol..

[28] Pedersen S.E., Hurd S.S., Lemanske R.F., Becker A., Zar H.J., Sly P.D., Soto-Quiroz M., Wong G., Bateman E.D. (2011). Global strategy for the diagnosis and management of asthma in children 5 years and younger. Pediatr. Pulmonol..

[29] Labbok M.H., Starling A. (2012). Definitions of breastfeeding: Call for the development and use of consistent definitions in research and peer-reviewed literature. Breastfeed. Med..

[30] Soto-Ramirez N., Karmaus W., Zhang H., Davis S., Agarwal S., Albergottie A. (2013). Modes of infant feeding and the occurrence of coughing/wheezing in the first year of life. J. Hum. Lact..

[31] Raoof N.A., Adamkin D.H., Radmacher P.G., Telang S. (2016). Comparison of lactoferrin activity in fresh and stored human milk. J. Perinatol..

[32] Ahrabi A.F., Handa D., Codipilly C.N., Shah S., Williams J.E., McGuire M.A., Potak D., Aharon G.G., Schanler R.J. (2016). Effects of extended freezer storage on the integrity of human milk. J. Pediatr..

[33] Lawrence R.A. (1999). Storage of human milk and the influence of procedures on immunological components of human milk. Acta Paediatr. Suppl..

[34] Nam S.H., Seo Y.M., Kim M.G. (2010). Bisphenol a migration from polycarbonate baby bottle with repeated use. Chemosphere.

[35] Robinson L., Miller R. (2015). The impact of bisphenol A and phthalates on allergy, asthma, and immune function: A review of latest findings. Curr. Environ. Health Rep..

[36] Ogbuanu I.U., Karmaus W., Arshad S.H., Kurukulaaratchy R.J., Ewart S. (2009). Effect of breastfeeding duration on lung function at age 10 years: A prospective birth cohort study. Thorax.

[37] Tully M.R., Lockhart-Borman L., Updegrove K. (2004). Stories of success: The use of donor milk is increasing in North America. J. Hum. Lact..

[38] Reddel H.K., Bateman E.D., Becker A., Boulet L.P., Cruz A.A., Drazen J.M., Haahtela T., Hurd S.S., Inoue H., de Jongste J.C. (2015). A summary of the new GINA strategy: A roadmap to asthma control. Eur. Respir. J..

[39] Miller M.R., Hankinson J., Brusasco V., Burgos F., Casaburi R., Coates A., Crapo R., Enright P., van der Grinten C.P., Gustafsson P. (2005). Standardisation of spirometry. Eur. Respir. J..

[40] Van Meel E.R., de Jong M., Elbert N.J., den Dekker H.T., Reiss I.K., de Jongste J.C., Jaddoe V.W.V., Duijts L. (2017). Duration and exclusiveness of breastfeeding and school-age lung function and asthma. Ann. Allergy Asthma Immunol..

[41] De Benedictis F.M., Bush A. (2017). Infantile wheeze: Rethinking dogma. Arch. Dis. Child.

[42] Wenzel S.E. (2012). Asthma phenotypes: The evolution from clinical to molecular approaches. Nat. Med..

[43] Zedan M.M., Laimon W.N., Osman A.M., Zedan M.M. (2015). Clinical asthma phenotyping: A trial for bridging gaps in asthma management. World J. Clin. Pediatr..

[44] Ek W.E., Karlsson T., Hernandes C.A., Rask-Andersen M., Johansson A. (2018). Breast-feeding and risk of asthma, hay fever, and eczema. J. Allergy Clin. Immunol..

[45] Kramer M.S., Chalmers B., Hodnett E.D., Sevkovskaya Z., Dzikovich I., Shapiro S., Collet J.P., Vanilovich I., Mezen I., Ducruet T. (2001). Promotion of breastfeeding intervention trial (PROBIT): A randomized trial in the republic of belarus. JAMA.

[46] Kramer M.S., Matush L., Vanilovich I., Platt R., Bogdanovich N., Sevkovskaya Z., Dzikovich I., Shishko G., Mazer B., Promotion of Breastfeeding Intervention Trial Study G. (2007). Effect of prolonged and exclusive breast feeding on risk of allergy and asthma: Cluster randomised trial. BMJ.

[47] Flohr C., Henderson A.J., Kramer M.S., Patel R., Thompson J., Rifas-Shiman S.L., Yang S., Vilchuck K., Bogdanovich N., Hameza M. (2018). Effect of an intervention to promote breastfeeding on asthma, lung function, and atopic eczema at age 16 years: Follow-up of the probit randomized trial. JAMA Pediatr..

[48] Asher I., Pearce N. (2014). Global burden of asthma among children. Int. J. Tuberc. Lung Dis..

[49] Kramer M.S. (2011). Breastfeeding and allergy: The evidence. Ann. Nutr. Metab..

[50] Kramer M.S. (2009). Methodological challenges in studying long-term effects of breast-feeding. Adv. Exp. Med. Biol..

[51] Leung J.Y., Kwok M.K., Leung G.M., Schooling C.M. (2016). Breastfeeding and childhood hospitalizations for asthma and other wheezing disorders. Ann. Epidemiol..

[52] Da Costa Lima R., Victora C.G., Menezes A.M., Barros F.C. (2003). Do risk factors for childhood infections and malnutrition protect against asthma? A study of Brazilian male adolescents. Am. J. Public Health.

[53] Vehling L., Chan D., McGavock J., Becker A.B., Subbarao P., Moraes T.J., Mandhane P.J., Turvey S.E., Lefebvre D.L., Sears M.R. (2018). Exclusive breastfeeding in hospital predicts longer breastfeeding duration in Canada: Implications for health equity. Birth.

[54] Miliku K., Voortman T., Bakker H., Hofman A., Franco O.H., Jaddoe V.W. (2015). Infant breastfeeding and kidney function in school-aged children. Am. J. Kidney Dis..

[55] Thulier D., Mercer J. (2009). Variables associated with breastfeeding duration. J. Obstet. Gynecol. Neonatal Nurs..

[56] Wen X., Kong K.L., Eiden R.D., Sharma N.N., Xie C. (2014). Sociodemographic differences and infant dietary patterns. Pediatrics.

[57] Frisell T., Oberg S., Kuja-Halkola R., Sjolander A. (2012). Sibling comparison designs: Bias from non-shared confounders and measurement error. Epidemiology.

[58] Smith G.D., Ebrahim S. (2004). Mendelian randomization: Prospects, potentials, and limitations. Int. J. Epidemiol..

[59] Lowe A.J., Carlin J.B., Bennett C.M., Abramson M.J., Hosking C.S., Hill D.J., Dharmage S.C. (2006). Atopic disease and breast-feeding––Cause or consequence?. J. Allergy Clin. Immunol..

[60] Dogaru C.M., Strippoli M.P., Spycher B.D., Frey U., Beardsmore C.S., Silverman M., Kuehni C.E. (2012). Breastfeeding and lung function at school age: Does maternal asthma modify the effect?. Am. J. Respir. Crit. Care Med..

[61] Dong G.H., Qian Z.M., Liu M.M., Wang D., Ren W.H., Bawa S., Fu J., Wang J., Lewis R., Zelicoff A. (2013). Breastfeeding as a modifier of the respiratory effects of air pollution in children. Epidemiology.

[62] Woodward A., Douglas R.M., Graham N.M., Miles H. (1990). Acute respiratory illness in Adelaide children: Breast feeding modifies the effect of passive smoking. J. Epidemiol. Community Health.

[63] Montgomery S.M., Ehlin A., Sacker A. (2006). Breast feeding and resilience against psychosocial stress. Arch. Dis. Child.

[64] Beasley R., Crane J., Lai C.K., Pearce N. (2000). Prevalence and etiology of asthma. J. Allergy Clin. Immunol..

[65] Beasley R. (1998). Worldwide variation in prevalence of symptoms of asthma, allergic rhinoconjunctivitis, and atopic eczema: ISAAC. Lancet.

[66] Kelly Y.J., Watt R.G., Nazroo J.Y. (2006). Racial/ethnic differences in breastfeeding initiation and continuation in the United Kingdom and comparison with findings in the United States. Pediatrics.

[67] Albernaz E., Araujo C.L., Tomasi E., Mintem G., Giugliani E., Matijasevich A., Onis M., Barros F.C., Victora C.G. (2008). Influence of breastfeeding support on the tendencies of breastfeeding rates in the city of Pelotas (RS), Brazil, from 1982 to 2004. J. Pediatr..

[68] Brown A. (2017). Breastfeeding as a public health responsibility: A review of the evidence. J. Hum. Nutr. Diet..

[69] Gross T.T., Powell R., Anderson A.K., Hall J., Davis M., Hilyard K. (2015). WIC peer counselors’ perceptions of breastfeeding in African American women with lower incomes. J. Hum. Lact.

[70] Kent G. (2015). Global infant formula: Monitoring and regulating the impacts to protect human health. Int. Breastfeed. J..

[71] Dagher R.K., McGovern P.M., Schold J.D., Randall X.J. (2016). Determinants of breastfeeding initiation and cessation among employed mothers: A prospective cohort study. BMC Pregnancy Childbirth.

[72] Mirkovic K.R., Perrine C.G., Scanlon K.S. (2016). Paid maternity leave and breastfeeding outcomes. Birth.

[73] Geraghty S.R., Khoury J.C., Kalkwarf H.J. (2005). Human milk pumping rates of mothers of singletons and mothers of multiples. J. Hum. Lact..

[74] D’Alessandro A., Scaloni A., Zolla L. (2010). Human milk proteins: An interactomics and updated functional overview. J. Proteome Res..

[75] Arrieta M.C., Stiemsma L.T., Dimitriu P.A., Thorson L., Russell S., Yurist-Doutsch S., Kuzeljevic B., Gold M.J., Britton H.M., Lefebvre D.L. (2015). Early infancy microbial and metabolic alterations affect risk of childhood asthma. Sci. Transl. Med..

[76] Waidyatillake N.T., Dharmage S.C., Allen K.J., Lodge C.J., Simpson J.A., Bowatte G., Abramson M.J., Lowe A.J. (2018). Association of breast milk fatty acids with allergic disease outcomes—A systematic review. Allergy.

[77] Doherty A.M., Lodge C.J., Dharmage S.C., Dai X., Bode L., Lowe A.J. (2018). Human Milk Oligosaccharides and Associations with Immune-Mediated Disease and Infection in Childhood: A Systematic Review. Front. Pediatr..

[78] Oddy W.H., Rosales F. (2010). A systematic review of the importance of milk TGF-beta on immunological outcomes in the infant and young child. Pediatr. Allergy Immunol..

[79] Smilowitz J.T., Lebrilla C.B., Mills D.A., German J.B., Freeman S.L. (2014). Breast milk oligosaccharides: Structure-function relationships in the neonate. Annu. Rev. Nutr..

[80] Ballard O., Morrow A.L. (2013). Human milk composition: Nutrients and bioactive factors. Pediatr. Clin. N. Am..

[81] Peroni D.G., Pescollderungg L., Piacentini G.L., Rigotti E., Maselli M., Watschinger K., Piazza M., Pigozzi R., Boner A.L. (2010). Immune regulatory cytokines in the milk of lactating women from farming and urban environments. Pediatr. Allergy Immunol..

[82] Munblit D., Boyle R.J., Warner J.O. (2015). Factors affecting breast milk composition and potential consequences for development of the allergic phenotype. Clin. Exp. Allergy.

[83] Miliku K., Goruk S., Becker A.B., Padmaja S., Mandhane P., Turvey S.E., Lefebvre D.L., Sears M.R., Field C.J., Azad M.B. Human milk fatty acids: Associations with maternal characteristics and infant body composition in the child study. Proceedings of the Canadian National Perinatal Research Meeting.

[84] McGuire M.K., Meehan C.L., McGuire M.A., Williams J.E., Foster J., Sellen D.W., Kamau-Mbuthia E.W., Kamundia E.W., Mbugua S., Moore S.E. (2017). What’s normal? Oligosaccharide concentrations and profiles in milk produced by healthy women vary geographically. Am. J. Clin. Nutr..

[85] Sinanoglou V.J., Cavouras D., Boutsikou T., Briana D.D., Lantzouraki D.Z., Paliatsiou S., Volaki P., Bratakos S., Malamitsi-Puchner A., Zoumpoulakis P. (2017). Factors affecting human colostrum fatty acid profile: A case study. PLoS ONE.

[86] Su L.L., S K.T.C., Lim S.L., Chen Y., Tan E.A., Pai N.N., Gong Y.H., Foo J., Rauff M., Chong Y.S. (2010). The influence of maternal ethnic group and diet on breast milk fatty acid composition. Ann. Acad. Med. Singap..

[87] Brenna J.T., Varamini B., Jensen R.G., Diersen-Schade D.A., Boettcher J.A., Arterburn L.M. (2007). Docosahexaenoic and arachidonic acid concentrations in human breast milk worldwide. Am. J. Clin. Nutr..

[88] Urwin H.J., Miles E.A., Noakes P.S., Kremmyda L.S., Vlachava M., Diaper N.D., Perez-Cano F.J., Godfrey K.M., Calder P.C., Yaqoob P. (2012). Salmon consumption during pregnancy alters fatty acid composition and secretory iga concentration in human breast milk. J. Nutr..

[89] Van Elten T.M., van Rossem L., Wijga A.H., Brunekreef B., de Jongste J.C., Koppelman G.H., Smit H.A. (2015). Breast milk fatty acid composition has a long-term effect on the risk of asthma, eczema, and sensitization. Allergy.

[90] Hoppu U., Isolauri E., Laakso P., Matomaki J., Laitinen K. (2012). Probiotics and dietary counselling targeting maternal dietary fat intake modifies breast milk fatty acids and cytokines. Eur. J. Nutr..

[91] Bianco-Miotto T., Craig J.M., Gasser Y.P., van Dijk S.J., Ozanne S.E. (2017). Epigenetics and DOHaD: From basics to birth and beyond. J. Dev. Orig. Health Dis..

[92] Martino D., Prescott S. (2011). Epigenetics and prenatal influences on asthma and allergic airways disease. Chest.

[93] Hartwig F.P., Loret de Mola C., Davies N.M., Victora C.G., Relton C.L. (2017). Breastfeeding effects on DNA methylation in the offspring: A systematic literature review. PLoS ONE.

[94] Tao M.H., Marian C., Shields P.G., Potischman N., Nie J., Krishnan S.S., Berry D.L., Kallakury B.V., Ambrosone C., Edge S.B. (2013). Exposures in early life: Associations with DNA promoter methylation in breast tumors. J. Dev. Orig. Health Dis..

[95] Rossnerova A., Tulupova E., Tabashidze N., Schmuczerova J., Dostal M., Rossner P., Gmuender H., Sram R.J. (2013). Factors affecting the 27k DNA methylation pattern in asthmatic and healthy children from locations with various environments. Mutat. Res..

[96] Karlsson O., Rodosthenous R.S., Jara C., Brennan K.J., Wright R.O., Baccarelli A.A., Wright R.J. (2016). Detection of long non-coding RNAs in human breastmilk extracellular vesicles: Implications for early child development. Epigenetics.

[97] Alsaweed M., Hartmann P.E., Geddes D.T., Kakulas F. (2015). Micrornas in breastmilk and the lactating breast: Potential immunoprotectors and developmental regulators for the infant and the mother. Int. J. Environ. Res. Public Health.

[98] Azad M.B., Konya T., Persaud R.R., Guttman D.S., Chari R.S., Field C.J., Sears M.R., Mandhane P.J., Turvey S.E., Subbarao P. (2016). Impact of maternal intrapartum antibiotics, method of birth and breastfeeding on gut microbiota during the first year of life: A prospective cohort study. BJOG.

[99] Forbes J., Konya T., Guttman D.S., Field C.J., Sears M.R., Becker A.B., Scott J.A., Kozyrskyj A.L., Azad M.B., the CHILD Study Investigators (2018). Formula exposure in hospital and subsequent infant feeding practices: Associations with gut microbiota and overweight risk in the first year of life. JAMA Pediatr..

[100] Bode L., McGuire M., Rodriguez J.M., Geddes D.T., Hassiotou F., Hartmann P.E., McGuire M.K. (2014). It’s alive: Microbes and cells in human milk and their potential benefits to mother and infant. Adv. Nutr..

[101] Kaplan J.L., Shi H.N., Walker W.A. (2011). The role of microbes in developmental immunologic programming. Pediatr. Res..

[102] Moossavi S., Miliku K., Sepehri S., Khafipour E., Azad M.B. (2018). The prebiotic and probiotic properties of human milk: Implications for infant immune development and pediatric asthma. Front. Pediatr..

[103] Dethlefsen L., McFall-Ngai M., Relman D.A. (2007). An ecological and evolutionary perspective on human-microbe mutualism and disease. Nature.

[104] Abrahamsson T.R., Jakobsson H.E., Andersson A.F., Bjorksten B., Engstrand L., Jenmalm M.C. (2014). Low gut microbiota diversity in early infancy precedes asthma at school age. Clin. Exp. Allergy.

[105] Pabst H.F., Spady D.W., Pilarski L.M., Carson M.M., Beeler J.A., Krezolek M.P. (1997). Differential modulation of the immune response by breast- or formula-feeding of infants. Acta Paediatr..

[106] Beijers R., Riksen-Walraven J.M., de Weerth C. (2013). Cortisol regulation in 12-month-old human infants: Associations with the infants’ early history of breastfeeding and co-sleeping. Stress.

[107] Wright R.J. (2008). Stress and childhood asthma risk: Overlapping evidence from animal studies and epidemiologic research. Allergy Asthma Clin. Immunol..

[108] Waidyatillake N.T., Allen K.J., Lodge C.J., Dharmage S.C., Abramson M.J., Simpson J.A., Lowe A.J. (2013). The impact of breastfeeding on lung development and function: A systematic review. Expert. Rev. Clin. Immunol..

[109] Hill A.B. (2015). The environment and disease: Association or causation? 1965. J. R. Soc. Med..

[110] Asher M.I., Keil U., Anderson H.R., Beasley R., Crane J., Martinez F., Mitchell E.A., Pearce N., Sibbald B., Stewart A.W. (1995). International study of asthma and allergies in childhood (ISAAC): Rationale and methods. Eur. Respir. J..

